# Painting psychosis: an empirical investigation of the self-portraits of Edvard Munch

**DOI:** 10.3389/fpsyt.2025.1643264

**Published:** 2026-02-05

**Authors:** Eric C. Bettelheim, Jingyi Liu, Paola Dazzan, Federico Turkheimer

**Affiliations:** 1 Department of Neuroimaging, King’s College London, London, United Kingdom; 2 Department of Neuroimaging, King’s College London School of Neuroscience, London, United Kingdom

**Keywords:** psychosis, schizophrenia, Edvard Munch, painting, self-portraits, neuroscience of art, stylistics, quantitative methods

## Abstract

Substantial evidence demonstrates that the brain is more interested in faces than in other subjects and that self-related material, particularly self-images, have higher saliency than non-self-referential material. Studies of self-portraits have revealed correlations between stylistic elements and artists’ states of mind. Edvard Munch, a founder of Expressionism and most famous for “The Scream”, was pre-occupied with depicting his subjective experience and a prolific painter of self-portraits. He has been posthumously diagnosed as suffering from schizophrenia, anxiety, bipolar and other disorders associated with altered perception. Munch’s painted self-portraits were empirically examined to determine if variations in stylistic elements, contrast, colour and fractal dimension, correlate with life events associated with psychopathology. His portraits were also examined as controls and to test whether images of others, related and unrelated to him, vary stylistically from his self-portraits and from each other. Productivity was examined as an independent indicator. Significant changes in contrast, colour, fractal dimension and productivity during critical periods in his life were identified in his self-portraits consistent with the conclusion that Munch is diagnostically best described as suffering from early onset psychosis. Examination of his portraits of related and unrelated people revealed differences from self-portraits and from each other consistent with comorbid social anxiety disorder.

## Background

The brain is more interested in faces than other subjects and in self-images more than non-self-referential material (1–3). Uniquely in art, in self-portraits the subject and object are the same. During their creation three images of the artist are involved: i) the face in the mirror or photograph, ii) the internal self-representation and iii) the image on a surface. The artist’s gaze, internal and external, never leaves the self. This constitutes a unique feedback system engaging visual perception, self-reflection and motor activity suggesting that self-portraits are more likely to reveal the artist’s state of mind than other artworks. In previous work, we have used quantitative tools for the analysis of self-portraits to infer pathophysiological traits from the production of artists with known mental health disturbances but not clear pathophysiology. Hence we have demonstrated a strong association of increased contrast in in the self-portraits of Vincent van Gogh during periods of extreme absinthe- consumption, a liquor popular with the artistic crowd in Paris in the 1860s but also a potent GABA interneuron inhibitor likely to generate psychotic episodes in subject with schizophrenic traits (4). We have also demonstrated the use of colour intensity (e.g. reds and yellows), in the self-portraits of Frida Kahlo during episodes of anger and pain (5). Others have similarly used fractal dimension (an indicator of healthy neuronal activity) in paintings to reveal reduced complexity in prominent aging artists suffering from forms of dementia (6) and increased complexity in particular periods of artist’s careers associated with behavioural change (7). More broadly, impairment in visual processing and spatial orientation in their art has been found in patients suffering from schizophrenia and obsessive compulsive disorder and in artists with psychosic experience (8, 9). Studies have revealed relationships between perceptual distortion in patients suffering from schizophrenia and artistic creativity suggestive of expression of emotional states (10).

Disturbances in the sense of self have been associated with various disorders including both depression (morbid pre-occupation) and schizophrenia (loss of boundaries) since the foundations of psychiatry (11–13). In schizophrenia these disturbances extend to self-perception (14, 15). ‘Signe du mirroir’, the inability to recognise, or a sense of strangeness when regarding, oneself in the mirror has long been recognised as characteristic (16–18). Sufferers often complain of a sense of disintegration of themselves and of their environment and of vivid, often overwhelming, sensory (19) and particularly visual, experiences (20, 21).^1^ The Scream’ can be understood as a depiction of such an event.^2^ Recent research has identified numerous morphological and functional deficiencies in the visual systems of those suffering from schizophrenia (25, 26) and its frequent comorbidities (27) of depression (28–30), and alcohol (31) and nicotine (32, 33) abuse, which provide a neurobiological foundation for such experiences (26, 34–36)‘. 

Edvard Munch (1863-1944) ‘Munch’ lived in the era in which the foundations of modern psychiatry were being laid (37–40) and he and his circle were aware of these developments (41, hereafter, ‘Prideaux’). Diagnosis, while at an early stage, was recognizably in accordance with modern constructs but not contemporaneously applied to him. Munch has been retrospectively diagnosed with a variety of disorders including schizophrenia (42), bipolar disorder (BPD) (43–45), narcissistic personality disorder (46, 47), borderline personality disorder (48), anxiety of various kinds including agoraphobia (49, 49, 50) and depression (46, 51). This illustrates both the complexity of his condition and the difficulty of post-mortem diagnosis even if relatively recent and where, as here, there is voluminous documentation (52–54)^3^.

## Biography – critical events

Munch is regarded by many as the father of Expressionism a school of art focused on conveying the artist’s subjective experience particularly using colour and distortion (59, 60). Although Munch initially painted in a naturalistic manner this began to change with the ‘The Sick Child’ an image of his older sister dying of tuberculosis. He later referred to it as his ‘breakthrough’ (61, p. 184; hereafter, ‘Holland’). This change in style became consolidated in 1889 after the death of his father. Emerging from his grief he wrote his ‘St Cloud Manifesto’ in which he committed himself to no longer painting “interiors, people who read and women who knit” but instead, “living people who breathe and feel, suffer and love” (62, p. 60; hereafter, ‘Tøjner’).

His determination to convey his own feelings in a new style became manifest in paintings of Despair (1892), Melancholy (1892), the Scream (1893), Anxiety (1894) and Jealousy (1895). Loneliness, illness, death, and bitter erotic experience became themes which continued to preoccupy him throughout his life.^4^ Munch was keenly aware of the role physical and psychological illness played in his life and considered both essential to his art (63).^5^ This may explain why he resisted treatment, like other creative people, until he had a complete breakdown in 1908 (43). He saw his art as essentially confessional, a form of autobiography and of self-therapy (64, hereafter, ‘Ustvedt’, 57). This is reflected in the number of self-portraits he painted, 51, in addition to 20 lithographs, including his first, and over 100 other works on paper (65–67).

Munch was the second of five children born within six years to an army physician and a considerably younger tubercular mother. At the time of his December birth his parents were living in rented rooms in a rural farmhouse. In his first year the family moved to the then rapidly industrialising capital city of Kristiana (now Oslo). Although from a prominent family, Munch’s father had a modest income which declined as he established his practice in poor areas of the city causing the family to move several times as it approached poverty. This trend worsened after the death of Munch’s mother from tuberculosis when he was 5. His older sister, to whom he was deeply attached, died of the same disease when he was 14. Tuberculosis was so common in Norway at the time that it was known as the national disease (68, 69). In 1912, near its peak, 80% of children in Kristiana were infected by the time they started school (70).

At birth Munch was so ill with respiratory disease that his father made arrangements for his immediate baptism and burial (Prideaux). Munch suffered respiratory illness throughout his life on several occasions requiring hospitalisation. He was unable to attend school for extended periods due to ill health spending entire winters confined to bed and ultimately could not continue. At the time of his mother’s death and again during an episode of tubercular-like infection age 13, he experienced vivid religious hallucinations. He blamed himself for the death of his sister in the following year as she had nursed him. In addition to his mother and sister the disease was responsible for the deaths of his maternal grandmother and maternal aunt. Despite his chronic vulnerability he survived pneumonia and the Spanish Flu ultimately dying of bronchitis in old age.

There is evidence of mental illness in Munch’s family. His paternal grandfather died in an asylum.^6^ One of Munch’s younger sisters had symptoms of schizophrenia in adolescence and spent most of her adult life in an institution. His father, a lifetime smoker who came from a devout family, became depressed following his wife’s death and expressed increasing religious obsession and dependence on alcohol. His father’s preoccupation with divine punishment in the form of disease and death was such that Munch’s childhood hallucinations were terrifying audible and visual images of skeletons, angels, and devils there to draw him into death (Prideaux). He often woke in the middle of the night wondering whether he was in hell (Tøjner).

Munch therefore had multiple reasons for emotional distress. His respiratory disease presented a constant, life-long threat of death, embodied in the deaths of close relatives, an anxiety reinforced by his father’s religious preoccupations. It imposed repeated periods of social isolation compounded by frequent changes in household. Soon after birth his mother put him in her sister’s care. After this initial separation he then experienced the death of his mother and older sister to whom he attached himself in the aftermath. His aunt, who he suspected of wanting to marry his father and replace his mother, became the only adult on whom he could rely although she too was tubercular (58; Tøjner). Unlike his father, who strongly disapproved, she recognised and nurtured his artistic talent and supported his ambition to be an artist (71). Despite her life-long attention and affection Munch kept her at a distance even unwilling to attend her funeral (Prideaux).

Against his father’s wishes Munch left technical college to pursue an artistic career attending art school for a year and then seeking informal instruction from various local artists. He adopted a bohemian lifestyle led by local writers and political radicals. During this time his abuse of alcohol and tobacco became apparent (Prideaux; 72). His first exhibition in 1885 attracted the attention of the local art establishment which provided him with the means to study in Paris. He postponed the trip due to ill health and had his first love affair with a woman of his mother’s age, the wife of a cousin, a military physician like his father. The experience filled him with what can be reasonably seen as Oedipal guilt and shame expressed in a painting called “Ashes”.

The end of the affair precipitated a period of mental disturbance which included heavy drinking and smoking, vertigo, agoraphobia, despair and jealousy (Prideaux). By year-end he was sufficiently recovered to visit Paris then at the height of the Impressionism wave; a style he adopted for a time. On his return to Kristiana, he began work lasting over a year on ‘The Sick Child’ exhibited in 1886. To his dismay, given the importance of the subject to him and the intense effort he had made, it was heavily criticised by critics and the public. Munch was so discouraged he left Norway beginning two decades of voluntary exile first in France in 1889 and from 1892 in Germany. While in France he was virtually destitute, often going without food, drinking heavily, visiting brothels and gambling unsuccessfully (Heller. Prideaux, Stang).

In 1889 he suffered near fatal pneumonia and in 1890 received news of his father’s death. He moved away from his bohemian circle and became deeply depressed. Soon thereafter he was again hospitalised with pneumonia and suffered a nervous collapse. Still unrecognised as an artist he continued to drink heavily often painting for days without rest (Prideaux). An exhibition in Berlin in 1892 so disturbed the conservative establishment that it was immediately closed down. The resulting ‘succes de scandale’ made him famous and attracted the attention of patrons and critics. In 1893, he recalled and painted the hallucinatory experience depicted in The Scream in which the figure and the environment seem to be dissolving.

“The air turned to blood – with cutting veins of flame….That shrill, bloody red. On the road and the fence. The faces of my comrades became a garish yellow-white. I felt a huge scream welling up inside me – and I really did hear a huge scream. The colours in nature broke the lines in nature. The lines and the colours quivered with movement. These vibrations of light caused not only the oscillation of my eyes. My ears were also affected and began to vibrate. So I actually heard a scream. Then I painted The Scream” (Tøjner, p. 96).

In 1894 his younger sister was institutionalised in an asylum near the bridge depicted in The Scream (Prideaux). In 1896 his only brother died of pneumonia age 30, shortly after his marriage, confirming Munch’s fear of marriage, association of female intimacy with death and compounding his sense of loss (73; Stenersen; Tøjner). His difficulty in maintaining relationships and intense social anxiety^7^ led to frequent impulsive travelling both to escape intimate contact, particularly with women, and to pursue commercial opportunities (Prideaux; Stang). His sometimes intense friendships did not last long. He could not tolerate the physical presence of people for any length of time including family members with whom he maintained life-long attachments (Heller; 74, hereafter ‘Howe’; Prideaux).

During this period Munch displayed a wide variety of symptoms including panic, phobias, insomnia, somatic complaints, pressured speech, strange ideas, hallucinations, delusions and paranoia. The latter was evident on several occasions when he provoked others violently. His brawls with strangers were notorious. On at least four occasions he used a firearm. He had three love affairs but never established a stable intimate relationship relying on prostitutes, servants and models for sexual experience. Munch’s abuse of alcohol was so severe that he was the subject of concern by his contemporaries (Ustvedt). He sought respite in various sanatoria^8^ for a variety of somatic complaints including insomnia, gastrointestinal and cardiac distress, vertigo, limb numbness and phobias including agoraphobia and fear of beds which he associated with illness and death (Tøjner). During this time he also took various drugs including bromides, aspirin and medication for the heart, fever and rheumatism (Prideaux).

He experienced increasing paranoia, convinced that others were speaking disparagingly of and plotting against him. In 1902 a struggle with an obsessive lover involving a revolver resulted in the loss of part of a finger on his left hand. He depicted her several times as Charlotte Corday and himself as the murdered Marat encapsulating both his anxiety about women and his association of sex and death. His condition deteriorated and in 1908, experiencing vivid auditory and visual hallucinations and paranoid delusions, he was admitted to a clinic in Copenhagen where he remained for 9 months. On admission he was diagnosed as having dementia paralytica due to alcohol poisoning (Heller; Prideaux; Tøjner).

He was initially kept sedated and then received mild electric treatment, massage and scented baths but the ‘therapy’ essentially consisted of enforced abstinence, regular meals and rest. He became sober and remained largely abstinent thereafter. In a self-portrait painted just before his release he appears to be emerging from dissolution. In 1909 he returned to Norway having received a national honour and the public recognition he had long sought. He purchased rural property outside of Oslo and thereafter he lived in isolation “just like a monk” (318; Prideaux; Stang, p. 222; Stenersen). He was unable to tolerate the presence even of close family members (Prideaux). Several housekeepers and models with whom he had sexual relations were tolerated but only so long as those relations continued.

He occasionally allowed patrons who had commissioned portraits to visit, but none were allowed to stay the night (Stenersen). He worked in an eccentric fashion talking constantly which he described as, “a defence” preventing others from speaking and their words entering and taking him over. He glanced at his subjects and then painted his first impression. Sitters often moved or left as he continued to talk and paint. He famously said, ‘I paint not what I see but what I saw” (Heller p. 42; Prideaux; Tøjner). This was his modus operandi; to capture not the detail of the image but its immediate impact on him (Ustvedt).

Although awarded large state and private commissions and accumulating increasing wealth he lived an ascetic life. His style noticeably changed from his pre-treatment period and included landscapes and local people. He surrounded himself with copies of portraits he had painted which he referred to as his “guardians”, “children” and his “family”. He sometimes “punished” them by leaving them outside or banishing them to an upstairs storage room (Stenersen). When indoors he continually played a radio broadcasting in languages he did not understand, or simply white noise, to drown out other sounds and exclude intrusive invisible waves which he believed encircled the world. The house was kept illuminated as he was frightened of the dark. It was surrounded by barbed wire, the gates padlocked when he was present and he kept fierce dogs. He continued to suffer insomnia, have delusions and feelings of persecution, particularly by the tax authorities, until his death which he insisted be unattended (Stenersen; Tøjner).

During his lifetime heavy drinking, and promiscuous ‘bohemian’ behaviour was common among artists (75; Prideaux; 76) yet despite this and his myriad symptoms, Munch was a productive and innovative artist throughout his life including during periods when he sought recovery in sanitoria and clinics. While there is some evidence of cognitive impairment, he was capable of successfully pursuing his commercial self-interest in a complex and rapidly evolving art market. Beginning his career in relative poverty and heavily criticised he died wealthy and internationally recognised (65; Heller; Prideaux; Stenersen).

### Somatic illness

Munch’s chronic respiratory illness brought him near to death on at least four occasions. Although never diagnosed it seems likely that he suffered from congenital tuberculosis which has non-specific presentation including respiratory distress and chronic lung disease, and it mimics a variety of conditions including pneumonia (77, 78). Transmitted by the mother it can cross the blood brain barrier and cause persistent neuroinflammation (79). Tuberculosis is a heterogenous disease often characterised by remission in untreated adults and closely associated with anxiety, depression, psychosis and somatic disorders including insomnia (80–82). Although physicians attending Munch would have been familiar with tuberculosis maternal transmission was only medically described in 1935 (83).

### Mental illness

Some observers have suggested that Munch suffered from bipolar disorder (BPD) (43, 44); others incline toward schizophrenia as a diagnosis (42). Both are plausible depending on which aspects of behaviour and which periods of Munch’s life are the focus. Others have suggested disorders which encompass symptoms which can be comorbid with both schizophrenia and BPD but do not exclude either (McElroy 47, 49). Manic depression, now BPD, was distinguished from ‘dementia praecox’, now schizophrenia, 15 years before Munch was admitted to the Copenhagen clinic (40). This was well known to the clinic’s director and to other physicians Munch consulted but he was not diagnosed with either condition. His diagnosis on admission of dementia paralytica, usually associated with late stage syphilis, caused by alcohol poisoning was to a degree correct but no underlying cause of Munch’s alcohol dependence nor other source of his psychological distress was identified (Prideaux).

Both BPD and schizophrenia have hereditability rates above 50% (84–86) and are associated with both developmental and environmental factors (87–89) including maternal infection (90, 316), childhood trauma, and parental loss which are additive to the risk of psychosis (91–93). Loss of the mother in childhood increases the odds of psychosis by a factor of 2 (94). Loss of a sibling is comparable to the loss of a father (95). Loss of two first-degree relatives in childhood compounds the risk (96). Both BPD and schizophrenia are characterised by high rates of insomnia (97), comorbid abuse of alcohol (98, 99) and nicotine (100), and risky behaviour including gambling (101) and promiscuity (102). Munch’s life history includes all of these elements.

BPD I typically manifests in adolescence and early adulthood and diagnosis requires a manic episode not attributable to substance abuse preceded or followed by hypomanic or major depressive episodes. Mania is characterised by elated mood, persistent goal directed activity, accompanied by decreased sleep, pressured speech, racing or distracted thoughts or high risk activities. BPD 1 also must include social or occupational dysfunction, hospitalisation or psychosis. Given Munch’s hallucinations and delusions BPD II is ruled out. The absence of manic symptoms when abstinent militates against both BPD I and cyclothymic disorder (103, hereafter ‘DSM 5’) as do the childhood onset of hallucination, the persistence of delusions in adulthood as well as the experience of olfactory hallucination (104; Stenersen).

Munch’s life history reveals virtually all known risk factors and phenomenology of schizophrenia other than motor dysfunction (DSM 5; 105, 106), and all of the predicates and elements of social anxiety (107).^9^ In addition to the factors mentioned above, he was born in winter (110) to an older father (111) and tubercular mother (112, 113) suffering immediate physiological (81) and psychological trauma (114), moved in his first year to an industrial city (115–117), experienced childhood audio-visual hallucination (118), experienced separation from his mother at birth, on her death and then on the death of his sister (119; Prideaux) the latter accompanied by intense feelings of guilt and shame (120) and social isolation due to his chronic respiratory disease (121, 122; Prideaux). In adulthood, prior to becoming abstinent, he evidenced somatisation and conversion disorder, the presence of physical symptoms, including limb numbness, gastrointestinal and cardiovascular distress, sensory or motor dysfunction where there is no apparent neurological or physiological cause, which characterises two-thirds of schizophrenia patients (123, 124).

Disturbances in the dynamics of perception, such as hallucination, the experience of real perception where no external stimulus is present (125, 126) and delusion, fixed beliefs based on inadequate grounds, not amenable to a rational argument or evidence to the contrary, including persecutory delusions, are key elements of psychosis experienced by 70-80% of schizophrenia spectrum sufferers (127–129). Both are often comorbid with anxiety disorders (130) and may be understood as efforts to resolve perceptual incoherence and sensory misperception and to reduce anxiety (131, 132).

In delusion formation the intensity of perception is altered and inappropriately high levels of salience are attributed to sensory information which might otherwise be disregarded (39, 133). In paranoia visual information, including facial emotion and gestures, indicators of intention, are negatively biased, as is overheard speech. Threatening inferences are drawn leading to false beliefs and inappropriate behaviour (134–137) including violence and social isolation (138, 139).

The natural history of the untreated disease includes a longitudinal transition from early-stage prevalence of hallucination to chronic stage prevalence of delusion (129, 140). This appears to be related to changing dysregulation of neurotransmitters notably dopamine (141), but also involving glutamate (142), serotonin and GABA (143, 144). Childhood trauma is characterised by dopamine dysregulation, positive symptoms (145, 146) and is closely related to retinal dysfunction (34, 147) and morphological abnormalities (148).

### Visual perception

BPD is characterised by sharper, more accurate, sensory experiences including more accurate vision and hearing together with heightened states of empathy and awareness of others’ states of mind (149, 150). In schizophrenia, in contrast, there are visual deficiencies associated with functional and anatomical anomalies throughout the visual system including the occipital, temporal, parietal and prefrontal areas (25, 151–154) and throughout the ventral and dorsal pathways (26). Functionally, reduced visual acuity is widely observed; in one study approaching 70%; 60% of cases demonstrate visual disturbance of some kind. (155–158). Deficits include misperception of the size, distance (159), colour, brightness, contrast and contour of objects (160, 161), in processing spatial frequency 162), integration, binocular vision (163, 164), depth perception and re-visualisation (165) all of which are related to hallucination and delusion (156, 163, 166, 167).

Abnormality in retinal morphology and function are observed in children (168), adolescents (169, 170), young adults at high-risk (171, 172), and prodromal patients (173) and are so pervasive they may be endophenotypes of the disease (153, 174–177). Altered rod and cone electroretinogram (ERG) responses distinguish schizophrenia from other psychosis and from depression (158, 178–180). Optical coherence tomography (OCT) studies reveal reduced macular thickness and volume (181), thinning of both inner (bipolar, horizontal and amacrine cells) and outer (photoreceptor) layers (176) and reduced thickness in the retinal nerve fibre layer (RNFL) (167, 182, 183). Reductions in ganglion cell layer (GCL) and inner plexiform layer (IPL) thickness (184), enlarged optic cup volume, increased cup-to-disc ratios (185, 186) and related tissue loss are also characteristic (187–190). Inflammation and swelling of photoreceptors (191, 192) and microvascular abnormalities are also evident (167, 193) as are distinctive iris pigment dots and crypts related to altered colour vision and cognition (194). These abnormalities together with rod dysfunction appear to cause those with schizophrenia to experience colours as more vivid and are related to hallucinatory experience (148, 195).

Dysfunction at low spatial frequencies in the early stages of the disease progresses to dysfunction in higher spatial frequencies as the disease develops (174, 177, 196). Alterations in retinal morphology and visual processing appear correlated with cortical abnormalities and clinical symptoms (35, 197). Thinner retinal epithelium layer correlates with mania (189), reduced GCL and IPL volume are negatively correlated with disease severity (184), and there are non-linear associations between RNFL thickness and acute and chronic sufferers (198) and with negative and positive symptoms (199). Delusions correlate with deficits in contour integration, conceptual disorganization and perceptual instability (200).

### Contrast

Decreased contrast sensitivity and reduced contrast gain control are common in schizophrenia (160, 201–204). Deficits appear in first episodes and deteriorate as the disease progresses. (154, 205, 206). Both reduced contrast gain (207) and weaker surround suppression (208, 209) are suggestive of reduced inhibition in the visual cortex (210). Both hypo and hyper dopaminergic conditions can prevail in differing systems and at different points in disease progression (145, 211). Reduced dopamine levels in the retina lead to excessively strong coupling of horizontal and amacrine cells and to cone-amacrine cell dysfunction (212–214). This causes reduced contrast sensitivity and poorer colour vision (215–217) and loss of visual acuity (218–220). Lower contrast sensitivity at low spatial frequencies causes difficulty in discerning differing adjacent elements and the blurring of borders (221, 222). Spatial frequency processing, dependent on dopamine receptor function in the retina, is also dysregulated (223a; 224, 225).

While a large majority of studies of contrast perception in schizophrenia find an overall reduction in magnocellular function (196, 226, 227) studies of unmedicated sufferers reveal higher contrast sensitivity and hyperactivity in the pathway consistent with patient reports of vivid anomalous visual experience (228). Hypofunction may therefore be the result of antipsychotic medication (223, 229). However, medication does not explain reduced function in first degree relatives (168), prodromal (20), high risk and remitted patients (228) and in schizotypic subjects (221). Dysfunction at low spatial frequencies in the early stages of the disease progresses to dysfunction in higher spatial frequencies as the disease develops (174, 177, 196). The explanation appears to lie in the progression of the disease, characterized by retinal hyperfunction in the early stage, accompanied by hallucination and hypofunction, accompanied by delusion, as it develops (230). Moderate drinking impairs contrast sensitivity at low frequencies (231) and acute alcohol use reduces contrast sensitivity at all frequencies (232) as well as contrast gain affecting visual processing in both the magnocellular and parvocellular pathways (233, 234). This persists despite years of abstinence (235). Nicotine is associated with rod dysfunction (236).

### Colour

Colour perception dysfunction, increased intensity of, or change in perception, appears at all stages of schizophrenia (21, 36, 237–239). Elevated retinal dopamine leads to increased brightness and hyper-intense colour perception in first episode patients together with visual distortion (237, 240) consistent with reports from high risk and prodromal patients of “abnormal intensity of environmental stimuli, feelings of being flooded and inundated, and inability to focus attention to relevant details” (228, p. 183). Excess dopamine in the limbic system is associated with positive symptoms including hallucination and may be related to structural changes in the retina (189, 241). Colour vision impairment on the tritan (blue-yellow) spectrum is accompanied by retinal hypodopaminergic conditions (242). Alcohol reduces the capacity to distinguish colours increasing the recognition threshold across the colour spectrum. Impairment in perception of colour and luminance is found in young (243), chronic (244) and abstaining alcohol users (245). Reduced colour discrimination is also found in smokers consistent with cone dysfunction (32, 33, 246–249). These deficits in perception of contrast and colour are compounded in individuals on the schizophrenia spectrum who are also heavy drinkers and smokers (33, 238).

### Fractals

Mandelbrot (250) discovered that there is a random, chaotic element in the regularity of natural patterns which he called fractals. Fractals can be seen as emergent, non-linear, spatial and temporal patterns of both chaotic natural phenomena and of self-organising systems such as the brain, which can be measured empirically (251–253).^10^ This form of regularity can be seen to follow a log power law of fractal dimension ‘D’ varying from 1 to 2 or 2 to 3 depending on whether 2 or 3 dimensions are being measured. This variable can be seen as a measure of spatial order-disorder (number of regularities) and simplicity- complexity (number of elements). D increases as the overall structure increases in fine spatial detail reflecting the relative complexity of the object (250). The D value is essentially a ratio of filled, repeating structural detail to empty space. Fractals thus provide a statistically reliable method of quantifying visual complexity by measuring the relative amount of coarse and fine structure in any image (255–257) including art images (7, 258).

Features of the natural environment have fractal values in the mid-range of 1.3-1.5. Humans are attuned to these levels and experiments consistently demonstrate a human preference for fractal values in this range in landscapes, buildings, urban areas, artificial and biological forms as well as in art of all categories (259–261). Natural images with mid-range values are appealing (7), reduce stress (262) and stimulate calm attention (263, 264). Given its frequency in the natural world at all scales it is unsurprising that humans evolved to prefer the fractal mid-range and regard conformity with it as attractive and an element of beauty (262, 265–267). Artists unconsciously depict faces with fractal values in the mid-range (268, 269). Spatial frequencies in art significantly higher than the mid-range are associated with triggering epileptic seizure and migraine in susceptible individuals and aversion in healthy subjects (270).

Fractal analysis has illustrated changes in complexity which correspond to stylistically defined periods in artists’ careers (271). Jackson Pollack was found to have an identifiable pattern of fractal development in particular periods. Complexity in his work increased over time from a D value close to 1 to 1.72 in his ‘drip’ paintings significantly above the preferred middle range of 1.3-1.5 (7, 272, 273). This may account for early negative critical and public reactions (274). His contemporary Mondrian moved in the opposite direction from early naturalistic paintings with a fractal dimension of 1.7 to abstract paintings consisting of lines of varying thickness with fractal values approaching 1 (266, 275). Fractal analysis of the paintings of prominent artists has revealed that those suffering from neurological disorder in old age reveal declining D levels as compared with those of healthy artists which increase with age (6).

### Metrics

In summary, measuring average contrast tests rod and sensory/interneuronal function particularly depth perception. Use of colour is indicative of cone dysfunction as well as altered mood and fractal complexity reflects general cognitive organisation as each frequency reflects networks at different ranges (the faster frequencies the more local networks, the slower the distant ones).

### Hypotheses

It was hypothesised that longitudinal empirical analysis of elements of style in self-portraits would indicate the artist’s mental state and be distinct from portraits of others. In particular that contrast would decrease and that colour and fractal dimension would increase during periods of crisis and that these trends would be greater in self-portraits than in portraits. It was also hypothesised that portraits of others, related and unrelated, would vary significantly from self-portraits and from each other. It was predicted that, together with productivity, the results would be consistent with a diagnosis of schizophrenia with comorbid social anxiety.

## Material and methods

### Material

Munch’s extant work consists of 1789 paintings on canvas and wooden panels as well as over 7600 images on paper. He also produced approximately 260 photographs including self-portraits. Of his paintings on canvas and panel eight are known to have been lost, all of which he painted again and an additional 10 lost entirely. None of these were portraits or self-portraits. Of the total corpus of paintings 151 are executed entirely in media other than oil paint and excluded to ensure comparability. Of the remaining 1638 paintings in oil and oil mixed with other materials 51 are self-portraits; 3% of his total oeuvre. He painted self-portraits throughout his career beginning with his first exhibited painting and the last completed the year before his death (276). Included in this category are paintings in which his self-portrait is accompanied by another person; 120 self-portraits on paper are excluded (65).

Portraits examined here represent 13% of his oil paintings all of which are included except preparatory oil sketches and one for which no image is available. All of the portraits are oil on canvas, wood panel or cardboard and painted from life except four painted posthumously. The portraits include 33 images of family and friends. There are 18 images where two or more people are depicted ‘Group Portraits’ treated as single paintings two of which are included in the ‘Family and Friends’ category. Excluded are paintings of scenes in which figures may be identified as Munch, family members or friends but which lack faces or are not detailed enough to be regarded as portraits. Images of models are excluded except where included in a self-portrait. Munch often painted two versions of commissioned portraits one of which he kept for himself. In such circumstances both versions are included. The resulting study sample of 268 paintings, amounts to 16% of his total oil painted oeuvre.

There are few difficulties in authenticating Munch’s paintings. He kept most of his work which has remained in public ownership since his death. Paintings in the city of Bergen have been in public ownership since the original collector’s death. Paintings held elsewhere are well documented and their provenance is not in question. His entire extant painted oeuvre is presented in a catalogue raissone compiled by Woll (66). It is on this work that the present study relies for the paintings’ authentication and chronology.

### Methods

For present purposes the beginning of Munch’s career as an artist can be dated to 1880, the date of his first extant paintings. The following years of his life during which he was hospitalised, admitted to clinics, suffered severe privation or symptoms of physiological illness or psychological disorder which were recorded by him or others, and the years in which someone close to him died, were all identified from documentary and biographical sources and are here referred to as ‘critical years’. These were verified by review of multiple, independent source materials. All other years were deemed to be ‘non-critical’. All of Munch’s self-portraits and portraits were dated by year in which completed based on the catalogue raisonnè and categorised as being completed in a critical or ‘non-critical year’.

All of the images examined are high resolution (>300 PPI) digital images downloaded wherever possible directly from the institutions which own the original paintings. Where this was not possible high-resolution images were obtained from open sources. Most institutions provided images in Tag Image File Format (TIF or TIFF) which preserves image quality regardless of copying. Some images were provided in Portable Network Graphics (PNG) format which is equivalent to TIFF in all relevant respects. Museum TIFF and PNG image files are standardized as to colour The remaining images were obtained in high resolution non-standardised Joint Photographic Experts Group (JPEG) format. Images obtained in JPEG and PNG format were converted to TIFF using a publicly available on-line service (xconvert.com) to ensure both consistency and stability of the images during processing. Several images provided by institutions included picture frames or colour reference bars which were removed using Adobe Photoshop (adobe.com/uk). TIFF images were used to measure contrast and fractals and converted to JPEG to measure colour to meet software requirements.

The images were necessarily produced under various lighting conditions and using varying equipment which may have affected the results although there is reason to believe that this variation enhances overall reliability of results by reducing the likelihood of any one format biasing the data (277). The images had varying numbers of pixels which were not standardised as test results of all variables, contrast, colour and fractal dimension, using several resolutions, were found to be substantially the same after rounding. The images were analysed as a whole including the areas surrounding the face as no practicable and consistent method of isolating the face was found. Weighting of each variable was uniform throughout each image as differences in perception were presumed to affect the entire painting. Some portraits were deliberately weathered and others have altered due to age. Munch used only commercially produced pigments which vary consistently.

### Statistical approach

The principal empirical approach to the data is correlative. That is, to determine if changes in the variables, contrast, colour and fractal dimension, as well as productivity, correspond to critical events/periods in Munch’s life and if so in what way and if such changes are consistent with potential psychopathology. As only self-portraits (sample size 51), and portraits (sample size 268)^11^, as opposed to the entire oil painted oeuvre (1638), were examined the data points do not form a normal distribution nor, in the case of self-portraits, a large sample size, ruling out the use of common parametrical statistical tests such as ANOVA. The data is also dispersed and incomplete as there were years in which either no self-portraits or portraits were painted and years when multiple portraits or self-portraits were painted.

To deal with these inherent problems the principal focus was to compare the central values of each tested variable in non-critical and critical periods. In addition, non-parametrical statistical tests, Median and Mann-Whitney, were applied. The former tests whether the two medians (each variable in non-critical and critical years) are the same. Mann-Whitney tests whether the two distributions are the same. The Wilcoxon test was applied to test for changes in intervals including both non-critical and critical years.

The number of statistical tests applied may suggest the use of multiple comparisons corrections to avoid the finding of spurious results. However proper application of multiple comparisons procedures such as the Bonferroni correction becomes too conservative if the tests are correlated amongst each other both because the correlation is endogenous to the paintings (e.g. as in the case of application to different colour channels), or because it is exogenous (e.g. self-portraits and portraits are made by the same artist). Multiple comparison correction was not applied because the approach has inherently low power and measures are strongly correlated (so standard multiple comparison would overcorrect the risk of false positives). However this work can be seen as corroboratory of previous results by an author in regard to Vincent Van Gogh with similar pathophysiology (4) and therefore is strongly hypothesis driven and not exploratory, hence the much lower risk of false positives. There was no within painting normalisation in terms of edge definition, colour depth, and/or image smoothness given the absence of approaches that have been recognised as valid across the field. Appropriate normalisation of these parameters may reduce outcome variability.

### Contrast

**Figure 1:**
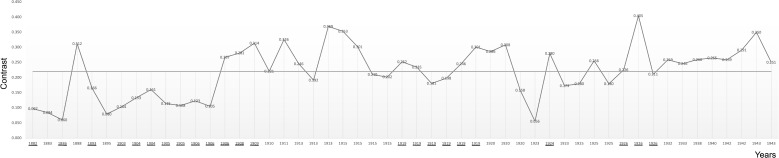
The plot shows the average contrast in Munch’s self-portraits in chronological order. Critical years are underscored.

**Figure 2:**
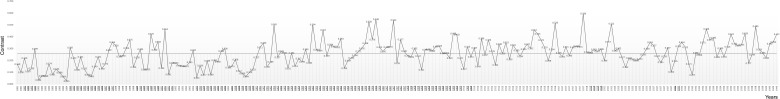
The plot shows average contrast in Munch’s portraits in chronological order. Critical years are underscored.

**Figure 3:**
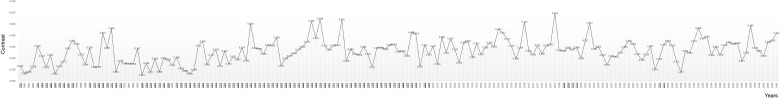
The plot shows the average contrast value in Munch’s portraits in chronological order excluding those of family and friends. Critical years are underscored.

To measure changes in levels of contrast an average measure of contrast was derived from each painting. In digital images the luminance of a pixel is derived from its red, green, and blue (RGB) wavelength components as 0.299R + 0.587G + 0.114B (*Digital Image Processing Using MATLAB, 3rd edition*). The images were analysed as a two-dimensional random process and the mean and autocorrelation function provided a value for the contrast of brightness. The autocorrelation function quantifies the average relationship between data points in a time series and their previous data points using the Wiener–Kinchin theorem (278). Matrix transformation of luminance values was conducted using the 2-D Fourier Fast Transform and the average spatial contrast was characterized by the value at origin (4). The calculations were performed using MATLAB (v. R2021a) resulting in the values on the y axis in **Figures 1**–**3**.

### Colour

**Figure 4:**
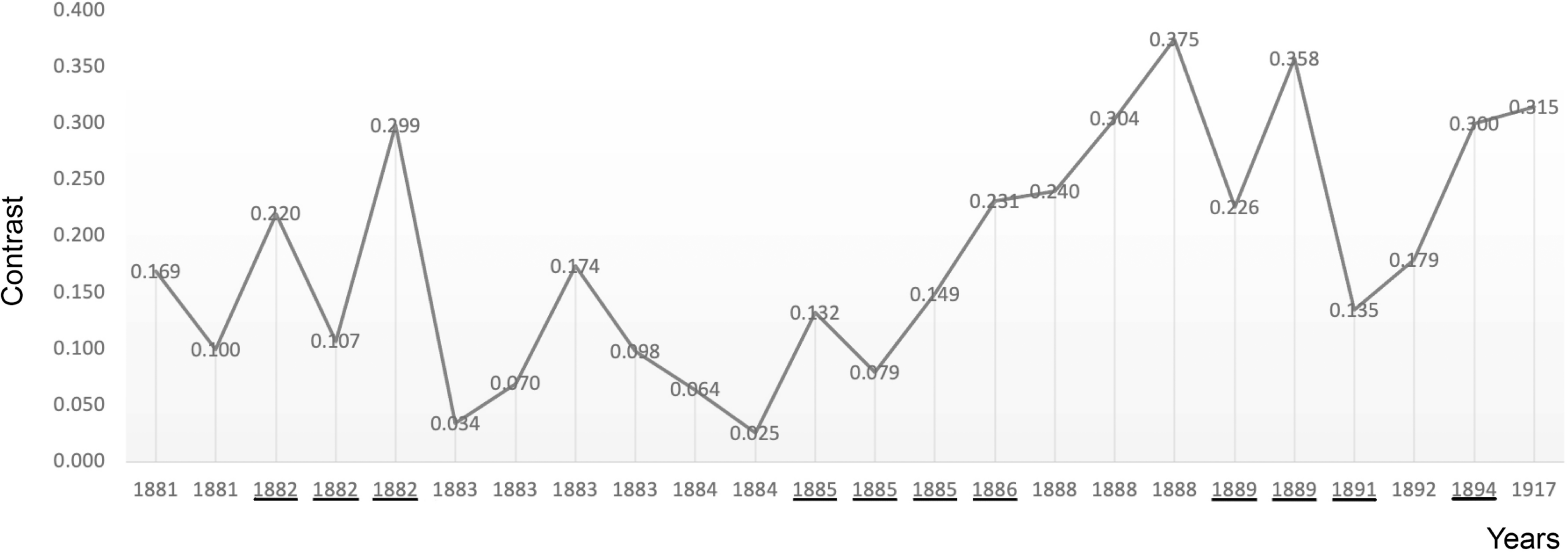
The plot illustrates the average levels of contrast in Munch’s portraits of family and friends in chronological order. Critical years are underscored.

**Figure 5:**
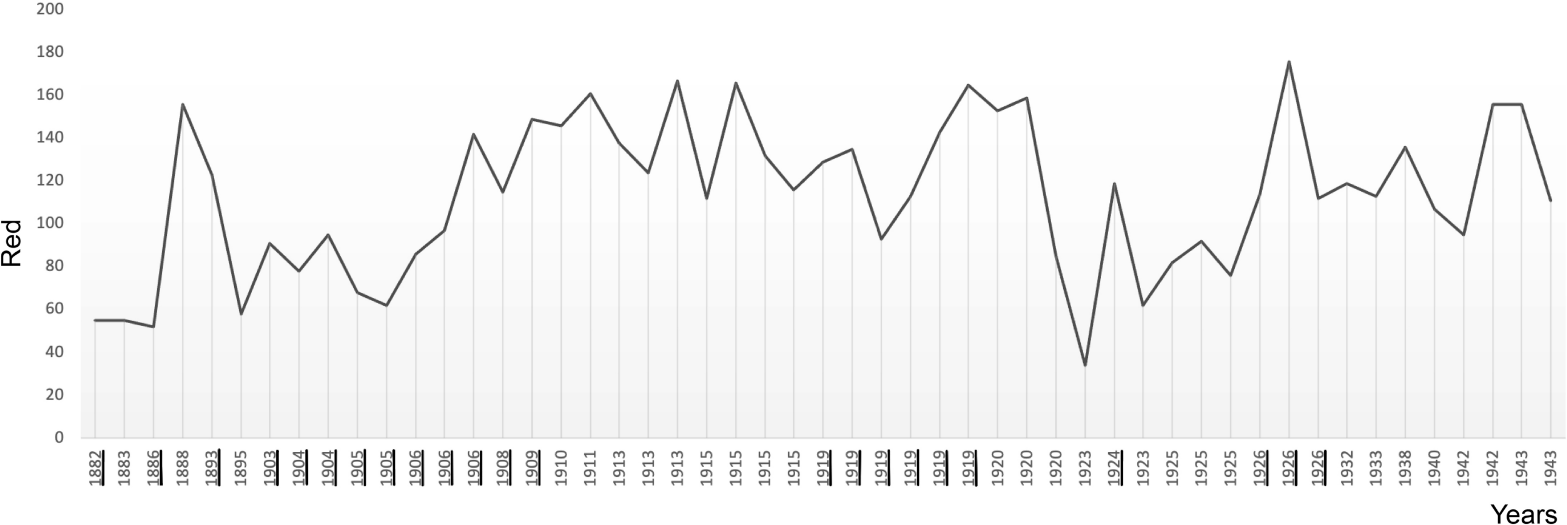
The plot shows the average use of red in Munch’s self-portraits in chronological order. Critical years are underscored.

**Figure 6:**
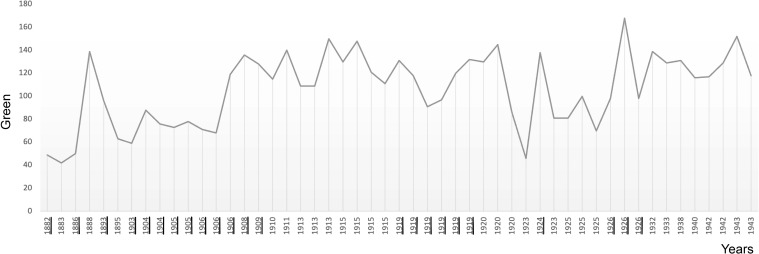
The plot shows the average use of green in Munch’s self-portraits in chronological order. Critical years are underscored.

**Figure 7:**
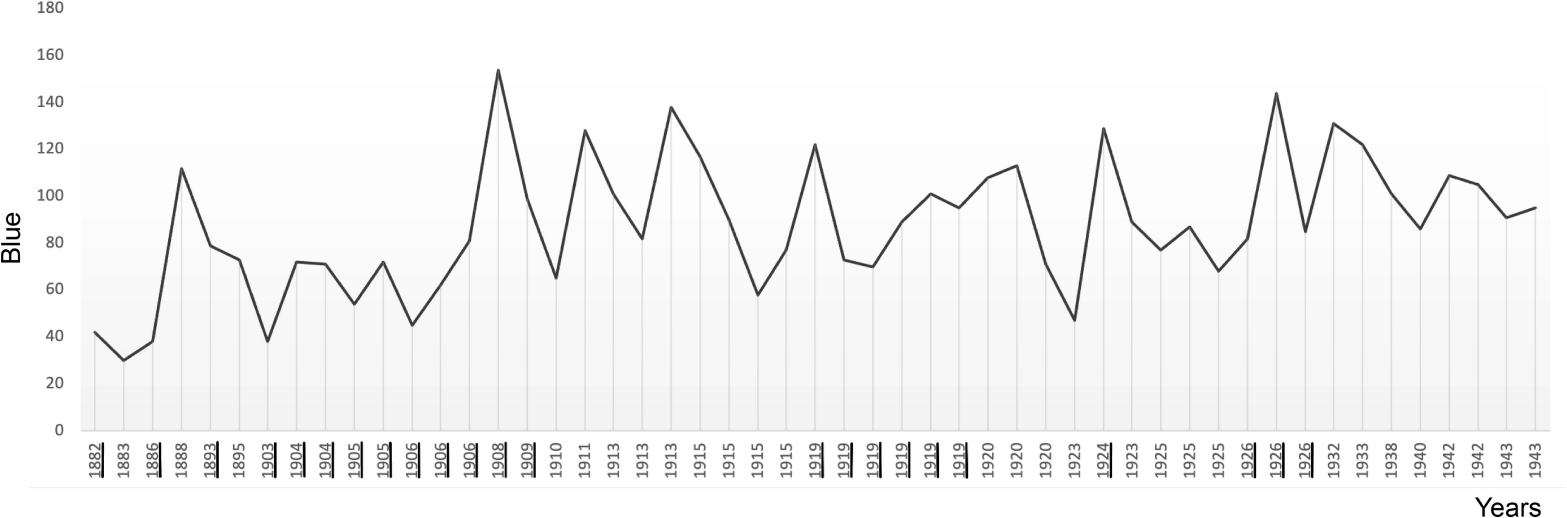
The plot shows the average use of blue in Munch’s self-portraits in chronological order. Critical years are underscored.

For each painting the colour median was measured in RGB and Cyan Magenta Yellow Black (CYMK) models. RGB, often known as additive colour mixing, is a method of encoding the three cone receptor wavelengths. In concept, each colour is made up of red, green, and blue light that shines with varying intensities. Hexadecimal coding allows integers ranging from 0 to 255 to be represent wavelengths plotted on the y-axis in **Figures 4**–**7**. The CMYK colour model is a subtractive colour model used in colour printing. It subtracts or masks colours from the white backdrop of the paper as ink decreases reflected light. The RGB and CMYK average values were calculated using MATLAB (v. R2021a) which applies consistent ranges of intensity in greyscale images to identify the specified colours.^^12^^

### Fractal dimension

There are two common methods of fractal analysis: one ‘2’ and one ‘3’ dimensional. Both utilise “box counting” a widely used sampling and data collection process. The fundamental approach is converting the image to greyscale and placing a sequence of grids of decreasing calibre (boxes) over a picture methodically and recording data for each subsequent calibre (counting) creating grids of progressively larger sizes. The 3-dimensional method has been selected here as considerable colour information is lost after conversion to greyscale which can affect the accuracy in roughness measurement.

**Figure 8:**
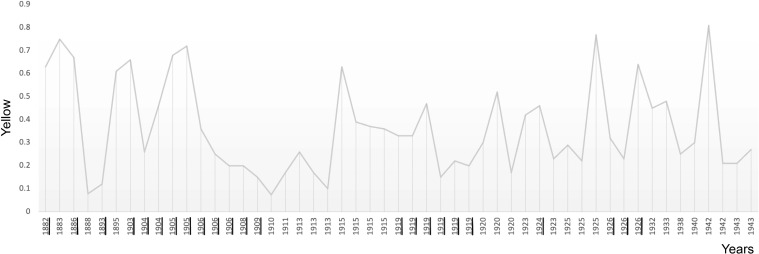
The plot shows the average use of yellow in Munch’s self-portraits in chronological order. Critical years are underscored.

**Figure 9:**
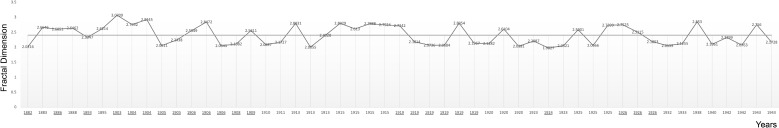
The plot shows the average fractal dimension of Munch’s self- portraits in chronological order. Critical years are underscored.

**Figure 10:**
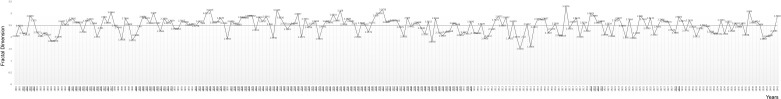
The plot shows the average fractal dimension of Munch’s portraits in chronological order. Critical years are underscored.

**Figure 11:**
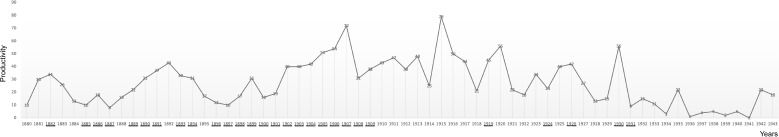
The table shows the number of oil paintings completed annually by Munch in chronological order. Critical years are underscored.

Every pixel of grayscale images has only one intensity channel; pixels of colour images have multiple intensity channels made up of red, green and blue. In order to reduce potential inaccuracy, a modified differential box counting approach (280) was used to determine fractal dimension values of RGB colour images. Combining the “probabilistic algorithm” method (281) and the “box merging” method extending box counting, (282) the fractal dimension of the resulting “3D” colour images was estimated using vectors in 5-dimensions (x, y, r, g, b). A greyscale image in this format generates 256 distinct shades of colour between 0 and 255. Applying this method to the 24-bit format for colour images resulted in individual RGB components calculated based on six possible combinations ranging from Ix,y (1) to Ix,y (6) which are averaged to improve accuracy of the roughness estimate. The range of D values using this method are between 2 and 3 which were plotted with log(S) on the x-axis and log(N(s)) on the y-axis in **Figures 8**–**11**. The midpoint of 3D analysis, 2.5, is equivalent to 1.5 in 2D analysis (283).

### Productivity

Productivity was measured by number of oil paintings completed in each year of Munch’s career as shown in **Figure 11**. Other works, including works in other media, works on paper and photographs were excluded due to a lack of reliable data as to dates of production.

## Results

### Contrast

Contrast analysis revealed a statistically significant positive correlation with age (Pearson R = 0.515. R^2 = 0.265, P<0.001). Overall lifetime average contrast declines in crisis years and more in the self-portraits than in the portraits (**Figures 1**, **2**, **Table 1**). Although, the Median and Mann-Whitney tests revealed no statistically significant relationship between critical years and contrast, the mean levels of contrast are noticeably higher in the portraits (Non-Critical Mean: 0.264; Critical Mean: 0.253; Hedges’ g: 0.104) than in the self-portraits (Non-Critical Mean: 0.241; Critical Mean: 0.204; Hedges’ g: 0.574). Analysis of the self-portraits in crisis periods reveals sharp increases in average contrast from previous levels and then declines as the period progresses. One can see an example of this trend in the period leading up to and during Munch’s stay in the Copenhagen clinic (1903-1909). There are notable singularities (point-change > 3 Std) in the years 1888, 1906, 1920, 1924 and 1926 where there are initial dramatic increases in contrast followed by declines. All of these occur during periods of crisis. There is also a notable increase in 1913 a non-critical year.

**Table 1:** The table compares contrast in Munch’s self-portraits (SP) and portraits (P) shown in **Figures 1**, **2** in terms of Range (R), Grand Median (GM), Non-Critical Median (NCM, Critical Median (CM), Median Test p-value (MT P), and Mann-Whitney p-value (M-W P).

	R	N-CM	CM	MT P	M-W P
SP Contrast	0.056-0.405	0.251	0.205	0.115	0.199
P Contrast	0.025-0.596	0.265	0.261	0.453	0.274

Analysis of the portraits reveals a similar pattern. There is again a statistically significant positive correlation of the average contrast with age (Pearson R = 0.400, R^2 = 0.16, P<0.001) with very similar quantitative values between the two sets and an identical rate of change: 0.003 units of positive change in contrast for every year. When the portraits are disaggregated it is noteworthy that unrelated individual portraits had the highest of all mean levels: Non-Critical Mean: 0.277; Critical Mean: 0.254; Hedges’ g: 0.226 (**Figure 3**) and that the mean levels of those of family and friends were the lowest of all: Non-Critical Mean: 0.173. Critical Mean: 0.201; Hedges’ g: 0.268 (**Figure 4**).

### Colour

Munch’s use of colour in his self-portraits also reveals a pattern of surges at the beginning of critical periods (**Figures 5**-**8**). Colour analysis reveals that Munch’s use of red and yellow tended to increase in periods of crisis and his use of blue decreases. There are statistically positive significant correlations of age with colours extracted with the RGB format. There was a positive correlation with Red (Pearson R = 0.303, R^2 = 0.091, P = 0.031; Hedges’ g: 0.059), Green, (Pearson R = 0.487, R^2 = 237 P<0.001; Hedges’ g: 0.201 and Blue (Pearson R = 0.440, R^2 = 0.194, P<0.001; Hedges’ g: 0.152). These changes likely underly a general increase in colour intensity (Pearson R = 0.413, R^2 = 0.17, P<0.001) but perceptual lightness, hue and saturation did not change with age. It is noteworthy that while red increased after 1906, its value was not sustained afterwards and correlation was weaker (did not survive multiple comparisons correction) which may indicate that red perception may be associated only with the acute phase of psychotic episodes. There were no associations of elements of the CMYK palette with age.

There are also similarly statistically positive significant correlations of age with colours extracted with the RGB format in the portraits. There was a positive correlation with Red (Pearson R = 0.378, R^2 = 0.143 P<0.001), Green, (Pearson R = 0.407, R^2 = 0.166, P<0.001) and Blue (Pearson R = 0.379, R^2 = 0.144, P<0.001). These changes likely underlay a general increase in colour intensity (Pearson R = 0.440, R^2 = 0.194, P<0.001) but again perceptual lightness, hue and saturation did not change with age. The portraits seem to follow the same general course as found in the self-portraits of increases in colour during critical periods followed by decreases in the RBG spectrum. Differently from the self-portraits, in the CMYK colour model all elements except K (black) were negatively associated with age. The largest correlation was a negative Pearson R correlation (R=-0.397, R^2 = 0.158, P<0.001) for Yellow. No statistically significant corelations were revealed by the Median or Mann-Whitney tests (see **Table 2**).

**Table 2:** The table summarises the results of examining average colour in Munch’s self-portraits (SP) and portraits (P) shown in **Figures 5**-**8**.

Munch SP	R	N-CM	CM	MT P	M-W P
Red	34-176	116	113.5	0.872	0.697
Green	42-168	117	97.5	0.568	0.254
Blue	30-154	90	80	0.196	0.278
Cyan	0.043-0.8	0.31	0.265	0.872	0.512
Magenta	0.076-0.81	0.3	0.295	0.872	0.864
Yellow	0.073-0.81	0.3	0.325	0.686	0.917
Munch P					
Red	18-229	131	132	0.944	0.96
Green	21-217	127	120	0.732	0.269
Blue	20-202	100.5	91	0.246	0.176
Cyan	0-0.94	0.4	0.36	0.195	0.991
Magenta	0.012-0.9	0.38	0.35	0.374	0.929
Yellow	0.02-0.9	0.375	0.35	0.634	0.935

Range (R), Non-Critical Median (NCM), Critical Median (CM), Median Test p-value (MT P), Mann-Whitney p-value (M-W P).

### Fractals

The median fractal value of Munch’s self-portraits rises in critical years (2.429; Hedges’ g: 0.259) (**Figure 9**). There are very significant results in both the Median (P<0.0001) and the Mann-Whitney tests (P<0.0001) of the portraits (**Table 3**) where the same pattern appears (Non-Critical Median: 2.392. Critical Median: 2.599; Hedges’ g: 0.680) (**Figure 10**). The Median test reveals greater increases in the self-portraits than in the portraits (**Table 3**) with portraits following the same pattern (Non-Critical Median: 2.295; Critical Median: 2.382) but at the lowest overall levels (**Figure 10**). Unrelated individual portraits, as with contrast, had the highest overall levels (Non-Critical Median: 2.42; Critical Median: 2.63, Hedges’ g: 0.690; data not shown).

**Table 3:** The table summarises the results of examining the average fractal values in Munch’s self-portraits (SP) and portraits (P) shown in **Figures 9**, **10**.

	R	N-CM	CM	MT P	M-W P
SP Fractal	1.983-3.071	2.33	2.423	0.903	0.608
P Fractal	1.512-3.308	2.423	2.624	0.0001	0.0001

Range (R), Non-Critical Median (NCM, Critical Median (CM), Median Test p-value (MT P), Mann-Whitney p-value (M-W P).

### Productivity

Munch was productive throughout his 60-year career with an annual average of 27 paintings (**Figure 11**). His median productivity during non-critical years (24.8) was 8% below the median (27.4) and 29% higher in critical years (31). The Generalized Linear model revealed a highly significant relationship between critical years and productivity (p<0.001). The most dramatic increase in productivity was in the period 1896 to 1909 reaching the second highest point in his career in 1907, the year before he entered the Copenhagen clinic. There were less dramatic but discernible rises in productivity in critical periods before, (1885-1895) and after, (1924-1926, 1930) his admission. In contrast, his productivity reached its highest point in 1915–16 a period of considerable success and large commissions. The other apparent exception, a drop in productivity in a period of crisis between 1936-1941, occurred when he was well into old age and suffered an embolism in one eye and deteriorating vision in both eyes. Nevertheless, once his vision recovered his productivity began to rise and he painted several important self-portraits before his death.

## Discussion

Munch was, from birth, a sick child; physically and mentally. The indications are that he inherited a genetic predisposition to schizophrenia and alcoholism and suffered maternally transmitted infection. Physiological trauma at birth was compounded by the psychological trauma of separation from his preoccupied mother; both must have been sources of intense anxiety. He depicted the additional trauma of witnessing his mother’s illness and death on numerous occasions. After his mother’s death he shifted attachment to his older sister who then died of the same disease that killed his mother. This further traumatic separation was compounded by his guilt that he had been the cause of her death. The impact of these deaths from tubercular-type illness, which his aunt suffered, as he did, was compounded by witnessing his father’s and his younger sister’s increasing mental disorder. It is thus not hard to see why he wrote:

“*I inherited two of humanity’s most dreaded enemies – consumption and mental illness. Sickness, madness and death were the black angels that surrounded my crib. My mother died prematurely – from her I inherited the seeds of consumption. My father was obsessively nervous and obsessively religious – to the point of madness. This had been the fate of his family for generations. From him I inherited the seeds of madness.*” (Tøjner p. 203-4).

The evidence is that Munch was correct in his self-diagnosis. His chronic illness, given its immediate onset upon birth, was very likely congenital tuberculosis. His mother, already ill when pregnant with him, both her sisters and likely his brother, died from tuberculosis. Munch’s life-long experience of respiratory illness in various forms is consistent with maternally transmitted disease. His genetic inheritance is indicated by a first degree relative, his sister, suffering from schizophrenia (92) and another, his father, evidenced depression (284), anxiety (285) in the form of obsessive religiosity (286, 287), and alcohol (288) and nicotine dependence (289), each of which entails a significant genetic component. Further, there is a bidirectional relationship between tuberculosis and psychosis (81, 290). It is certainly conceivable that this combination of transmitted illness and inherited predisposition can account for his condition.

Environmental and experiential factors are likely to have increased the likelihood of psychosis. Given the loss of not one but two primary attachments, his ambivalent relationship with his aunt, his difficulty in establishing relationships with women and association of female intimacy with death, it is reasonable to assume an insecure/anxious/avoidant attachment style which, together with trauma, is closely associated with psychosis and schizophrenia in particular (291–293). His inherited susceptibilities and the panorama of multiple developmental and environmental risk factors made childhood onset of psychosis highly probable (294). His social isolation due to his respiratory illness and the shame of his father’s religious preoccupation and disapproval of his choice of an artistic career and bohemian lifestyle, laid firm predicates for social anxiety.

Empirical, stylistic analysis of Munch’s self-portraits and portraits is consistent with perception in, and the natural history of, schizophrenia comorbid with social anxiety. Examination of contrast shows sudden increases at the start of critical periods then declines consistent with vivid episodes and reduced visual perception of contours (edges). There are a statistically significant positive correlations of average contrast with age both in the self-portraits and in the portraits with very similar quantitative values between the two. There is also an identical rate of change in contrast for every year. indicating a progressive deterioration of Munch’s condition as he grew older. Over time, the base levels of contrast increase consistent with deterioration in his untreated condition.

Colour use surges in critical periods in both self-portraits and portraits consistent with vivid psychotic periods. There are statistically significant positive correlations of age with colours extracted with the RGB format from the self-portraits indicating a general increase in colour intensity. The exception, a negative correlation for yellow, is an indication of dysfunction on the tritan axis consistent with dopaminergic dysregulation and psychosis. Colour evidence from the portraits, with both RGB and CYMK significantly increasing as he aged, is consistent with schizophrenia.

Fractal analysis reveals lower levels of complexity in non-critical years and higher levels in critical years in both the self-portraits and portraits, with markedly greater increases in the self-portraits. Greater complexity can be associated with more intense feelings. Munch’s productivity also increased significantly in critical periods commensurate with his own recognition that painting was a means of helping him to manage his emotions.^13^

When portraits are disaggregated, while following the same pattern between non-critical and critical years as the self-portraits, the levels of contrast, colour and fractal dimension are all lowest in related portraits and highest in unrelated portraits. This suggests not only greater distress in critical periods generally but heightened stress when painting strangers generally, consistent with comorbid social anxiety.

### Bipolar disorder

Aspects of Munch’s life history and some of his symptoms overlap with those of BPD 1 but many do not. If one focuses on the period between his first love affair and his treatment in Copenhagen Munch may be seen as suffering from BPD I with psychosis which occurs in approximately half of cases and is easily mistaken for schizophrenia (295). He would now not be diagnosed with BPD II given his psychotic experiences. BPD 1 typically becomes manifest in late adolescence or early adulthood not in childhood (296) by which time Munch had already experienced hallucination. Hallucinations can, albeit rarely, accompany BPD but do not persist and usually abate in the early years after onset (295, 297) and do not include olfactory hallucination (104). BPD is characterised by sharper, more accurate, sensory experiences including more accurate vision together with heightened states of empathy and awareness of others’ states of mind (149, 150). Munch’s portraits, consistent with reduced magnocellular function are characterised by a lack of detail, depth, and facial emotion; he ignored his sitters.

BPD’s natural course does include childhood anxiety and insomnia, but depression continues, regardless of periods of remission accompanied by long-term cognitive decline (298, 299). There are no reports by Munch or his contemporaries of low mood except as episodically related to episodes of guilt and grief nor is there any evidence of the widespread neurocognitive deficits, typical in BPD, in executive functioning, verbal and spatial memory and psychomotor performance, which usually persist in non-symptomatic periods (298, 299). While unable to function well socially, he remained in contact with a wide circle (59; Heller) and fully capable of managing his affairs including planning carefully for his death.

His periods of increased productivity, with one exception, were accompanied by negative feelings of guilt, shame and jealousy not the positive or euphoric feelings associated with mania and BPD. Mania is characterised by short bursts of goal directed activity, measured in days or weeks, not years-long periods of increased productivity (300). His reckless behaviour, violence, impulsive travelling and episodic depression all abated with abstinence. In his 35 year period of abstinence and isolation Munch does not report nor is he reported by contemporaries as appearing either manic or depressed although they did notice delusions and antisocial behaviour. His extensive journals and correspondence reveal self-absorption, sensitivity to criticism and obsessive concerns but neither the grandiosity of mania nor the futility of depression (61; Prideaux; Tøjner). There was no medication available so the absence of BPD symptoms during this period makes a diagnosis of BPD less plausible.

### Schizophrenia

Munch manifested all but one of DSM’s positive symptom of schizophrenia, deficient psychomotor behaviour, and his life history includes almost every known risk factor for the disease, genetic, developmental and environmental. The natural history of schizophrenia includes a shift from hallucination to delusion and changes in perception, accompanied, and probably caused, by commensurate dysregulation of neurotransmitters, dopamine particularly, in the retina and visual system generally. These are closely associated with deficits in perception of contrast and colour which are exacerbated by abuse of alcohol and tobacco. In Munch’s case the transition was associated with his becoming sober and choosing social isolation. The change in his behaviour was widely noted by contemporaries and the simultaneous change in subject matter and style has long been noted by art historians (Prideaux, Woll).

Such a transition, unaccompanied by depression or persistent or cyclical low mood, is indicative of schizophrenia accompanied by persecutory delusions and comorbid social anxiety rather than BPD. The correlations of increased contrast and colour with aging, particularly evident in the portraits, and the increasing fractal values in critical periods, suggest a long-term deterioration in his condition but not one accompanied by a declines in productivity or cognitive capacity. It is also noteworthy that a disrupted sense of self like that painted and described by Munch, distinguishes schizophrenia from BPD (301). Munch’s life thus parallels the natural history of schizophrenia: childhood pre-morbid and prodromal phases, first psychotic episodes, a florid psychotic phase, followed by stable delusions accompanied by negative symptoms and social deficits (230).

Although approximately 60% of schizophrenia patients express negative symptoms of blunted affect, anhedonia, alogia, avolition and asociality (302, 317). Munch appears not to have done so. Asociality, (a reduction in social initiative due to decreased motivation to form close relationships (303) seems to appear when he retreated to the countryside but does not fully account for his behaviour. Prior to that he sought out friendships, social contacts and public approval. There is little indication of other negative symptoms. Neither he nor his contemporaries report lack of affect, his emotional range was wide even exaggerated, and expressed in his art, writings and encounters with others. He had intense love affairs and an active sexual life into old age; his periods of depression and low mood prior to treatment were episodic and abated with sobriety; he was voluble, to the point of pressured speech, and articulate in both speaking and writing; and he never stopped producing new work and experimenting with media. The only dramatic declines in his productivity occurred at the time of his breakdown in 1908, he resumed work within a few months, and in old age when he suffered visual impairment and then too his productivity rose following recovery. His motivation to work persisted throughout his career. Indeed, as he recognised, work was essential to management of his emotions.

During the second half of his life he maintained, albeit from a distance, relations with his aunt and sisters who he supported financially, with a small circle of friends, lovers and patrons. These relationships were often difficult but his persistence in creating and maintaining them does not appear indicative of decreased motivation. His intolerance of their prolonged physical presence, together with other forms of anxious behaviour, including continued refusal to attend ceremonies celebrating him, despite his lifelong wish to be recognised and accepted by the public, and his continued sensitivity to criticism despite his success, appears to go beyond asociality as a negative symptom.

### Co-morbid social anxiety

Social anxiety disorder is characterised by anxious preoccupation with being judged negatively by others and avoidance of social and/or public performance situations due to an excessive concern with being humiliated and ashamed under the scrutiny of others (130, 304). It is now recognised as comorbid with schizophrenia in approximately 25% of cases, (305, 306) albeit overlapping with negative (and positive) symptoms which contributes to under-reporting (307, 309, 310). Comorbid patients’ avoidant behaviour is ego-dystonic differing qualitatively from anhedonia, or lack of social interest, are motivated, as Munch was, to gain control of their anxiety as opposed to lacking motivation to manage it (307).

His intense social anxiety, manifested prior to entering the Copenhagen clinic by impulsive travel and flight from intimate relations, continued after treatment in the extraordinary avoidance measures he employed which also appear related to his persecutorial delusions (308). It is noteworthy that when comorbid with schizophrenia social anxiety sufferers appear to present greater difficulty in mentalisation but less severe cognitive disturbance as compared with those without social anxiety (311). This fairly characterises Munch as recorded by his contemporaries and as evidenced by his voluminous correspondence. Munch had been increasingly isolated in childhood due to his illness, unable to attend school or complete higher education. When Munch abandoned the career chosen by his father in favour of an artistic one, his father condemned him publicly and privately causing Munch intense shame, a predicate for social anxiety (71). His first love affair was one of many occasions on which Munch felt deeply ashamed as was the exhibition of the Sick Child not least as blamed himself for his sister’s death He expressed deep feelings of shame, both in writing and in multiple works. He was intensely sensitive to criticism all his life avoiding exhibitions of his work and social occasions of all kinds including those celebrating his achievement (Prideaux). He often fled from personal, social and professional contacts (Stenersen). His need to avoid people worsened over time and was unaltered by treatment and abstinence. Munch’s anxiety about being judged negatively approached and at times crossed over into paranoia (312).

Comorbid sufferers are characterised by insecure attachment, childhood trauma, increased difficulty in mentalisation, a higher sense of shame, greater negative attribution bias and suicidality (304, 313). With the exception of suicidal planning, Munch demonstrates all these elements. He had multiple childhood trauma, disorganised and fearful attachments to his mother, sister and aunt, and a father who was over-controlling and shaming all of which are indicative of the social, generalised and phobic anxiety he experienced (314, 315). Contrast, colour and fractal values are all significantly higher in portraits of unrelated others in comparison to his self-portraits and portraits of those related to him, suggestive of his discomfort in the presence of strangers. This pattern of higher values in portraits is also reflected in his use of contrast and colour as he aged. This appears to confirm the greater difficulty in painting others, as opposed to himself and those close to him, throughout his life. It is also noteworthy that negative attribution bias is greater in comorbid patients (304). Munch never painted a happy face; his portraits and self-portraits all depict neutral or sad expressions.

## Conclusion

The findings here of stylistic variations in Munch’s self-portraits and portraits are consistent with the perceptual and emotional phenomena of early onset psychosis and comorbid social anxiety. BPD is inconsistent with the coincidence of alcohol abuse with mood disorder and reckless behaviour. His symptoms appear to follow the natural course of schizophrenia, shifting from hallucination to delusion. His anxiety, particularly social anxiety, like his insomnia, never abated. His painted self-portraits and portraits, which he saw, like all his painting, as a means of expressing himself. Statistically significant results reflect empirically the transitions in his state of mind: growing brighter, more colourful, more complex, and more numerous as his symptoms became more severe. This suggests that his childhood experience of both physiological and psychological distress caused him to acquire neurological traits including lasting morphological, neurobiological, and functional changes particularly affecting perception of contrast and colour. The rise in productivity during critical periods is consistent with his own understanding that his illnesses and his efforts to manage them, are reflected in his art.

## Limitations

The most important limitation of the present study is that it encompasses only a fraction of Munch’s paintings and none of his works on paper. Analysis of those works might reveal very different results. Second, the material examined are not the original paintings but digital images. These were produced under varying conditions and using a variety of equipment if to a largely consistent standard. This, at the very least, means that analysis of colour is of limited use given the difficulties which computers have of identifying colour in anything like the subtlety of the eye. Given the software employed the absence of standardized colour calibration will have generated a range of, as opposed to precise, colour values. There was no within painting normalisation in terms of edge definition, colour depth and or image smoothness given the absence of approaches that have been recognised as valid across the field. Appropriate normalisation of these parameters may reduce outcome variability Fractal and contrast analysis are more reliable but again could vary if applied to other and original works. No definitive diagnosis is possible based on such materials.

## Data Availability

The raw data supporting the conclusions of this article will be made available by the authors, without undue reservation.

## Footnotes

1 This strangeness also extends to auditory self-perception (22, 23).

2 Munch seems to have understood this. He wrote on the canvas: “*Kan kun være malet af en gal Mand!*” (“Can only have been painted by a madman!”) (24).

3 Munch left several thousand pages of journals, correspondence, poetry, and fiction. His exhibitions are well documented, there are numerous contemporary reviews of his art and a significant number of his contemporaries wrote memoires of him (for translated materials see: https://www.emunch.no/english.xhtml; for memoires see: 55, 56, Stang (57) and Stenersen, 1944 (hereafter, ‘Stenersen’ (58)).

4 For images of his work in chronological order:https://en.wikipedia.org/wiki/List_of_paintings_by_Edvard_Munch.

5 “My fear of life is necessary to me, as is my illness. Without anxiety and illness, I am a ship without a rudder. My art is grounded in reflections over being different from others. My sufferings are part of myself and my art. They are indistinguishable from me, and their destruction would destroy my art. I want to keep those sufferings.” (41, p.251; 62).

6 Although a consequence of spinal tuberculosis, it was a source of Munch’s belief in his hereditary madness (Prideaux; Tøjner).

7 “I can’t attend parties. Nobody understands. If I go to a party it has such a strong effect on me I simply cannot cope” (Prideaux, 295); “I feel like a prisoner when I attend parties and I don’t want to be anyone’s prisoner – my nerves can’t take it” (Stenersen,117).

8 Kornhaug 1899, Como 1901, Knock-su-Mer 1901, Eldursburdg 1904, Ilmenau 1905, Kosen 1906 (twice), Ilmenau 1906.

9 Social anxiety is the persistent fear of social situations threatening embarrassment or humiliation, which causes functional impairment in relationships with family and friends and in forming and maintaining romantic relationships (108; APA 2022b, 109).

10 The self can also be seen as fractal in structure (254).

11 Of the total portraits 33 are of ‘family and friends’ consisting of known relations and people, including some patrons, with whom he maintained relationships over several years.

12 For a detailed explanation of colour extraction using MatLab (see: 279).

13 “When I paint sickness and suffering it is…a healthy release. It is a healthy reaction from which one can learn and carry on living” (Tøjner, p.134).
